# Parkin as a Molecular Bridge Linking Alzheimer’s and Parkinson’s Diseases?

**DOI:** 10.3390/biom12040559

**Published:** 2022-04-09

**Authors:** Frédéric Checler, Cristine Alves da Costa

**Affiliations:** IPMC, UMR7275 CNRS-UCA, INSERM, Labex DistALZ, 660 Route des Lucioles, 06560 Valbonne, France

**Keywords:** parkin, PINK1, p53, XBP1s, Alzheimer’s disease, Parkinson’s disease, ER stress, autophagy, mitophagy, cell death, mitochondrial dysfunction

## Abstract

Alzheimer’s (AD) and Parkinson’s (PD) diseases are two distinct age-related pathologies that are characterized by various common dysfunctions. They are referred to as proteinopathies characterized by ubiquitinated protein accumulation and aggregation. This accumulation is mainly due to altered lysosomal and proteasomal clearing processes and is generally accompanied by ER stress disturbance, autophagic and mitophagic defects, mitochondrial structure and function alterations and enhanced neuronal cell death. Genetic approaches aimed at identifying molecular triggers responsible for familial forms of AD or PD have helped to understand the etiology of their sporadic counterparts. It appears that several proteins thought to contribute to one of these pathologies are also likely to contribute to the other. One such protein is parkin (PK). Here, we will briefly describe anatomical lesions and genetic advances linked to AD and PD as well as the main cellular processes commonly affected in these pathologies. Further, we will focus on current studies suggesting that PK could well participate in AD and thereby act as a molecular bridge between these two pathologies. In particular, we will focus on the transcription factor function of PK and its newly described transcriptional targets that are directly related to AD- and PD-linked cellular defects.

## 1. Alzheimer’s Disease in Brief

Alzheimer’s disease (AD) is the main age-related disease. Sporadic AD cases are characterized by a slow, progressive and irreversible degeneration, ultimately leading to mental disability and death [1,2]. The main macroscopic cerebral lesions consist in senile plaques, which are extracellular deposits mainly composed of amyloid β peptides (Aβ), and neurofibrillary tangles, which are intracellular lesions filled with hyperphosphorylated Tau proteins [3,4]. Additionally, exacerbated oxidative and ER stress/unfolded protein responses [5,6,7], alterations in mitochondrial structure, function and clearance (mitophagy) [8,9], autophagy [10,11], endolysosomal function [11,12,13,14] as well as synaptic loss [15,16,17] have been also observed at early stages in AD-affected brains. At later stages, although this has been discussed, apoptotic neuronal lesions and more particularly p53-dependent cell death have also been documented [18,19,20,21].

A few percent of AD cases are of familial origin and are due to mutations that segregate in an autosomal dominant manner [22,23]. The genes bearing these mutations have been identified and encode for the β-amyloid precursor protein (βAPP) and two family-related proteins referred to as presenilins 1 and 2 [24,25,26,27]. Genetic clues, anatomical stigmata and post-mortem tissue analyses have led to the proposal of the two main current etiological hypotheses. The hypothesis centered around Aβ, namely, the amyloid cascade hypothesis [28,29], proposes that Aβ accumulation is the early trigger of the degenerative process. This is strongly supported by the fact that mutations in βAPP (the precursor of Aβ) and in presenilins/γ-secretase (the enzyme releasing Aβ from βAPP [30,31]) affect the physiopathological processing of βAPP [32,33,34,35,36]. Alternatively, the tau-related hypothesis has been reinforced not only by the study of tau-related preclinical models [37] but also indirectly, by the consistent failure of pharmacological or immunological therapeutic strategies aimed at interfering with Aβ production or accumulation [38]. More recently, with the reconciliation of undoubtful genetic data and the failure of most Aβ-centric clinical trials, it has been proposed that additional proteases [39,40,41,42,43,44] and βAPP-related products distinct from Aβ could participate in the etiology of AD [40,41,45,46,47,48,49]. The latter postulate agrees well with the observations that some of these catabolites are indeed at the center of gravity of cellular dysfunctions observed early in AD [9,50,51,52].

## 2. Parkinson’s Disease in Brief

Parkinson’s disease is the second most frequent age-related pathology. PD is characterized by motor and non-motor symptoms [53,54,55]. It is a progressive and irreversible pathology characterized macroscopically by a drastic neuronal loss in the pars compacta of the substantia nigra, leading to an alteration in dopaminergic transmission [56,57,58,59]. At the histological level, PD is mainly characterized by intracellular inclusions named Lewy bodies that are mainly filled with aggregated α-synuclein [60,61]. PD is mainly idiopathic but there exist relatively rare genetic cases that could be due to either autosomal dominant or autosomal recessive transmission [62,63]. Monogenic forms due to a single mutation with Mendelian inheritance account for about 5–10 percent of PD cases.

More than fifteen chromosomal loci referred to as *PARK*s have been identified [64,65,66]. Of note, causative genes associated with a subset of loci are still pending definitive identification. Alternatively, the direct influence of some identified genes on the PD etiology remains to be definitely established. Confirmed genes responsible for the autosomal dominant form of PD have been identified as *PARK*1 (the *SNCA* gene encoding α-synuclein [67,68,69,70], *PARK*8 (*LRRK*2 [71]), *PARK*13 (*HTRA2* [72]) and *PARK*17 (*VPR35* [73]). Autosomal recessive genes include *PARK*2 (*Parkin* [74,75,76]), *PARK*6 (*PINK1* [77,78,79]), *PARK*7 (*DJ1* [80]) and *PARK*9 (*ATP13A2* [81]). Known functions of the above proteins clearly indicate that the etiology of PD is complex, although they point to ER stress [82,83], mitophagy [84,85], autophagy [86,87,88] and mitochondrial [89] and lysosomal [90,91,92,93] defects, as well as neuronal death exacerbation [94,95,96].

## 3. Alzheimer’s and Parkinson’s Diseases: Two Distinct Pathologies with Common Dysfunctions

### 3.1. Proteasomal Dysfunction

As stated above, AD and PD are proteinopathies, i.e., pathologies in which several generally misfolded proteins accumulate and are poorly cleared. On the whole, it has been reported that, whatever the nature of the aggregate components, they are all highly toxic [97]. This reduced propensity to proteolytic degradation is directly related to the drastic alteration of the ubiquitin–proteasomal system triggered by aggregates [98]. The proteasomal machinery, which underlies one of the major cellular degradative pathways, is altered in AD [99,100]. This conclusion has been supported by the observation of accumulated ubiquitin in both senile plaques and neurofibrillary tangles [101] and by the fact that proteasomal activity was reduced upon aging in an AD mouse model [102]. Indeed, it was demonstrated that Aβ peptides physically interact with the proteasome and inhibit its activity [103]. Accordingly, Aβ load is inversely correlated with proteasome activity in AD [104].

Several studies also reported proteasomal defects in PD [105,106] which lead to the disruption of α-synuclein clearance [107,108]. The key role of proteasomal degradation is indirectly supported by the fact that parkin acts as a ubiquitin-ligase [109] and promotes the degradation of proteins involved in mitochondrial structure [110] and autophagic processes [111] (see below).

### 3.2. Oxidative Stress Dysfunction

Aberrant production of oxidated proteins, lipids or nucleic acids and/or defects in cellular anti-oxidant defenses occurring during aging or in pathological conditions [112,113] have been consistently observed in AD and PD [114]. Proteins, lipids, DNA and carbohydrates are increased in the AD brain and advanced glycation end-products have been described in plaques [115]. Data concerning the cellular antioxidant portfolio, including several catalases, reductases and peroxidases, appear to show significant decreases in terms of both mRNA levels [116] and activities [117].

Excessive oxidative stress has also been documented in PD [118,119,120]. Particularly compelling was the recent systematic review indicating that oxidative stress markers could be recovered in cerebrospinal fluid and blood in PD-affected patients [120]. Of interest, this study reported a significant decrease in enzymatic and non-enzymatic antioxidants [120].

### 3.3. ER Stress and Unfolded Protein Response

The endoplasmic reticulum is where protein biogenesis and post-translational and structural modifications occur [121]. When neo-synthesized proteins are not correctly folded, they normally undergo proteasomal-mediated clearance via a cellular process referred to as ER-associated degradation (ERAD) [122,123]. This cellular program is linked to the unfolded protein response (UPR) that is directly initiated by ER stress [124] and involves three cellular pathways involving either pancreatic ER kinase (PERK1), activating transcription factor 6 (ATF6) or inositol-requiring enzyme-1 (IRE1α) [125]. Thus, in neurodegenerative diseases, accumulated toxic proteins could derive from combined exacerbated misfolding and proteasomal defects [126,127,128].

Activations of ER stress and UPR have been extensively documented in AD [6,7,17,129]. Thus, elevated ER stress response has been observed in APP/PS1 double transgenic mice as indicated by enhanced levels of GRP78 and phosphorylated forms of ERK and eIF2a [130]. Further, deletion of endogenous PERK prevents synaptic defects and cognitive alterations in APP/PS1 mice [131]. It should be noted that these alterations were not observed in APP Ki Tg mice that harbor a single mutation in APP [132]. However, in support of a genuine relationship between AD-related stigmata and ER stress/UPR alterations, it has been shown that intracerebral administration of Aβ42 alters the ER stress-associated proteins GRP78 and eIF2α in adult mouse brains [133]. Further, several studies reported ER stress-mediated effects of Aβ oligomers in various cellular models and AD brain samples [134,135,136]. Importantly, ruling out the possibility of an artifactual phenotype linked to overexpression, Aβ also accumulates in the ER and triggers UPR in iPSCs derived from sporadic and familial AD differentiated into neural cells [137]. Finally, it has been demonstrated that Tau blocks the ERAD pathway [138] and that the UPR is activated in pre-tangles in the AD hippocampus [5]. Although far from being exhaustive, these studies strongly suggest ER stress/UPR exacerbation in AD.

ER stress is also drastically altered in PD [83,139,140,141]. Several proteins responsible for familial PD have been documented as effectors of the ER stress machinery. α-Synuclein physically interacts with ER chaperones such as GRP78 [142] and phosphorylated α-synuclein perturbs ER–Golgi trafficking by altering several ER exit factors such as RAB1 and SNARES [143]. Further, in dopaminergic neurons, α-synuclein activates PERK and ATF6 pathways, while, conversely, GRP78 overexpression rescues α-synuclein-associated toxicity by elevating tyrosine hydroxylase and restoring dopamine levels in the substantia nigra [144].

ER stress also increases parkin protein and mRNA levels and the ER stress-mediated increase in parkin levels is prevented by ATF4 deletion in cells as well as in mice, likely because it functionally impairs ATF4 binding to the parkin promoter region [145]. Further, parkin overexpression protects against ER stress, while, conversely, parkin depletion sensitizes cells to ER stress [146]. It has been proposed that parkin could control ER stress response via its ubiquitin-ligase activity. Thus, Imai and colleagues showed that the parkin-associated endothelin receptor-like receptor (PAEL-R) behaves as a parkin substrate. An ubiquitinated insoluble form of PAEL-R occurs in familial PD-affected brains and its accumulation is prevented by parkin-mediated degradation [147].

XBP1s is a transcription factor that results from a stress-induced non-conventional splicing of XBP1 by Ire1α [148]. It has been recently demonstrated that there exists a functional interplay between XBP1s and PINK1. Thus, XBP1s activates the transcription of PINK1 in various cellular models, including primary cultured neurons, as well as in mouse brains [149], and thereby triggers a pro-mitophagic phenotype that was fully abolished by PINK1 depletion [149]. On the other hand, PINK1 was shown to phosphorylate XBP1s and modulate its transcriptional activity by governing its shuttling to the nucleus [149]. Of importance, PINK1-induced phosphorylation of XBP1s occurs at sites reminiscent of those phosphorylated in the substantia nigra of sporadic PD-affected brains [149].

A few papers concern LRRK2 that is mutated in autosomal dominant forms of PD (see above). LRRK2 is localized at the ER, suggesting a possible contribution to the control of ER stress response. Indeed, in *C-elegans*, LRRK2 protects dopaminergic neurons from 6-hydroxydopamine-associated toxicity, likely by promoting GRP78 enhanced expression [150].

### 3.4. Mitochondria Dysfunction

Most oxygen is consumed by mitochondria in the cytochrome oxygen chain. As stated above, in aging and neurodegenerative conditions, it is acknowledged that reactive oxygen species (ROS) formation is increased. Thus, there exists a loop wherein mitochondria are both producers and targets of ROS. In neurodegenerative conditions, it is therefore not surprising that reports have consistently indicated drastic alterations in mitochondrial structure and function. This has been particularly well documented in recent reviews [151,152,153,154].

Mitochondria dysfunctions are early and prominent alterations in the brains of patients with AD [9,155]. A bioinformatic analysis indicated that the mitochondrial oxidative phosphorylation system (OXPHOS) pathway was consistently altered in AD [156]. Particularly, a reduction of complex I of OXPHOS was observed in the hippocampus of AD-affected patients [157]. Recently, it was documented that mitochondria size alterations and cristae disorganization were observed together with increased ROS species production in AD cell models [9]. This was confirmed in various AD transgenic mice [9]. On the whole, AD is associated with mitochondrial defects that affect their biogenesis and dynamics, their transport, their contact with the endoplasmic reticulum [154,158,159] and their clearance by mitophagy (see below).

Mitochondrial dysfunction is also a feature of PD [89,160]. Multiple pathways directly related to mitochondria have been documented [161] that include increased ROS production [162], abnormally elevated intracellular calcium concentrations and reduced ATP formation [163,164,165]. Indeed, most of the proteins responsible for both autosomal and recessive cases of PD trigger drastic mitochondrial alterations. Amongst myriad works documenting these alterations, one could notice that DJ1 downregulation increases mitochondrial fragmentation and reduces mitochondrial potential [166]. LRRK2 regulates mitochondrial dynamics [167] and its pathogenic mutations impair the ability of MIRO to stop mitochondrial damage [168]. α-Synuclein is partly localized in mitochondria in PD-affected post-mortem brains [169] and disrupts mitochondrial function by increasing ROS [170]. Finally, the PINK1–parkin axis is recognized as the major system aimed at controlling mitochondrial homeostasis by mitophagy [171].

### 3.5. Autophagy/Mitophagy

Autophagy is a key cellular process by which cellular homeostasis is maintained. Mitophagy is an autophagic process that concerns the control of mitochondrial function and quantity. Homeostatic mitochondrial equilibrium between biogenesis and clearance by mitophagy is necessary for a wide spectrum of intracellular functions, including, among others, embryogenic development, cellular differentiation and death, as well as inflammation. In mammalian cells, it is admitted that the main pathway to regulate mitochondrial homeostasis involves PINK1 and parkin cooperation aimed at selectively clearing defective mitochondria [172,173].

As expected from the key role of mitochondria, mitophagy dysfunction is at the center of gravity of neurodegenerative diseases and mitophagy defects have been well documented in AD [174,175,176]. It has been shown that mitophagy reversed Aβ- and Tau-induced memory defects in *C. elegans* and that it lowered the load of insoluble Aβ40 and Aβ42 in a mouse model (APP/PS1) of AD [177]. Conversely, Aβ and phosphorylated Tau trigger defective mitophagy in an age-dependent manner, resulting in a severe augmentation of the number of mitochondria accompanied by a significant reduction in their mean size [178]. Besides canonical Aβ, additional fragments derived from APP have been shown to trigger drastic mitochondrial dysfunctions. Thus, the β-secretase-derived APP-CTF (C99) was recently shown to affect mitophagy in both cell and animal AD models, as well as in sporadic AD brains, as illustrated by mitochondrial size alteration and cristae disorganization in neuroblastoma cells [9]. Accordingly, C99 accumulation triggers mitophagy failure, as shown by the alteration of canonical proteins involved in mitophagy. Thus, enhanced conversion and accumulation of LC3, resistance to degradation of p62 and altered PINK1-mediated recruitment of parkin in mitochondria have been documented [9]. These cellular observations were confirmed in young 3xTg-AD mice (a widely used mouse model [179]) and in AAV-C99-infected mice [9]. These alterations were also reported in sporadic AD brain samples [9]. Of importance, a very recent study confirmed this set of data in induced neural stem cells (iNSCs) derived from AD-affected patient fibroblasts [180]. In iNSCs, C99 accumulates in mitochondrial domains and triggers mitophagy failure, as illustrated by increased LC3II and p62 proteins as well as by altered PINK1-mediated parkin recruitment to mitochondria [180].

As parkin and PINK1 are two proteins mutations in which account for a subset of autosomal recessive cases of PD, it is not surprising that mitophagy dysfunction is also a key cellular process affected in PD [84,85,181,182]. PINK1 accumulates at the outer membrane of dysfunctional mitochondria and recruits parkin which, in turn, ubiquitinates several proteins, including ubiquitin, the ubiquitination of which is central to autophagosome formation. This function in mitochondrial quality control results in poorly detectable levels of PINK1 due to a continuous import/degradation process. This virtuous circle is impacted in PD. A rate-limiting fission step in the mitophagy process implies that parkin–PINK 1 interplay should control the levels of inner and outer mitochondrial membrane proteins. In this context, it is noteworthy that mitofusins 1 and 2 are ubiquitinated in a parkin/PINK1-dependent manner [183,184] and that PINK1 displays the ability to phosphorylate dynamin-related-protein 1 (Drp-1) [185]. Several mechanistic explanations have been provided to link PINK1 with mitochondrial defects. One of the most common hypotheses proposes that increased fission and mitophagy could be accounted for by the ability of PINK1 to phosphorylate Protein kinase A. This kinase phosphorylates and activates Drp-1 at its serine 656 residue and a constitutively dephosphorylated Drp1 mutant (Ser- > Ala 656) triggers mitochondrial fragmentation and increased cell vulnerability [186]. Thus, a PD-linked loss of function of PINK1 could increase the dephosphorylated form of Drp-1 and thus increase fission and mitophagy [187,188]. Another interesting study indicated that, in contrast to previous anticipations, parkin could also act upstream to PINK1. Thus, Goiran and colleagues showed that parkin controlled PINK1 transcription via a FOXO3a-dependent mechanism in various transgenic and knock-out mice [189]. Thus, pathogenic mutations in both parkin and PINK1 could have a direct role in the alteration of the mitophagic process that takes place in PD.

### 3.6. Cell Death

Several types of cell death can occur. Necroptosis is a form of necrosis leading to unprogrammed cell death [190]. Programmed cell death, also named apoptosis, is a normal process that allows cellular homeostasis and the destruction of damaged cells. When apoptosis is exacerbated, neurodegeneration triggers neuronal loss and subsequent cerebral defects. The extent of apoptosis varies according to the brain pathology examined. It has been convincingly demonstrated in PD [95,191]. Among a bulk of studies, p53 has been documented as the prominent contributor to cell death in various degenerative diseases [20,192], including PD [193,194]. Thus, it has been shown that p53-like immunoreactivity is increased in PD-affected brains [195] and that p53 contributes to 6-hydroxydopamine-induced cell death, an experimental model of PD-like pathology [196,197,198]. Furthermore, selective depletion of p53 in dopaminergic neurons protects against neuronal loss [199]. It should be noted that most proteins responsible for familial cases of PD interact functionally with p53. α- and β-synucleins lower p53-dependent apoptosis [94,200,201], a phenotype abolished by 6-hydroxydopamine [108] while, p53 up-regulates α-synuclein transcription [202] and promotes its aggregation [203], thus underlining a functional interplay between α-synuclein and p53 [193]. Parkin can repress p53 transcription and this is abolished by PD-related mutations [204]. Further, parkin translocation to the nucleus and physical interaction with the p53 promoter are prevented by S-nitrosylation [205], a post-translational process exacerbated in PD-affected brains [206]. As is the case for α-synuclein, parkin also behaves as a transcriptional target of p53 [207,208]. DJ1 prevents p53-induced mitochondrial impairment [209] and is regulated in ER stress conditions by a molecular cascade involving parkin, p53 and the transcription factor XBP1s [210]. Finally, PINK1 behaves as a repressed transcriptional target of nuclear p53 [211], while, conversely, PINK1 can directly bind to and phosphorylate p53 and thereby control the autophagic process in hepatic cancer stem cells [212].

Apoptosis in AD is less consensual [213,214]. However as is the case with PD, p53 appears to play a key role in AD-related cell death stigmata [215]. Thus, p53 expression is increased in AD brains [216], correlates with the age of patients [217] and undergoes several AD-linked post-translational and conformational modifications [217,218]. Also important, in AD, apoptotic neurons display intracellular Aβ [19], which can regulate p53 promoter transactivation [219]. Further, there exists an intricate network of functional interactions between p53 and members of the γ-secretase complex [220]. Presenilins [221] and its presenilinase-derived C-terminal fragment [222] control p53-dependent cell death in AD. This phenotype is exacerbated by AD-related mutations [223]. Other components of the γ-secretase complex, namely, Pen-2, Aph-1 and nicastrin [224,225], all control p53-dependent cell death in either presenilins-dependent [226] or independent [227] processes. Finally, p53 also regulates the expression of βAPP [228]. Conversely, various APP-derived C-terminal fragments derived from γ-secretase [229] (AICD) or caspase 3 [230] (C31) cleavages can induce enhanced p53 promoter transcription [221,231] and translation [232] or toxicity [233,234].

## 4. Parkin, a Molecular Link between Alzheimer’s and Parkinson’s Diseases?

Parkin has been characterized as a pleiotropic protein involved in multiple cellular functions [76]. Since it was initially described as a ubiquitin-ligase [109] and thus as being committed to feeding the proteasomal machinery with ubiquitinated unfolded/misfolded proteins, parkin has been proposed as a key regulator of proteinopathies characterizing most neurodegenerative diseases [235,236]. Since parkin mutations are generally responsible for autosomal recessive familial cases of PD, particular attention has been paid to delineating parkin-associated dysfunction in this pathology.

However, several works have indicated that parkin could also contribute to AD. First, a macroscopic analysis of AD-related lesions indicated that parkin co-localized with both senile plaques and Aβ-related vascular lesions. Further, parkin expression also appears to be high in astrocytes surrounding both lesions [237]. These observations per se were obviously not sufficient to postulate a key role for parkin in AD, and the mechanistic explanation underlying these anatomical observations was initially lacking. According to the main ubiquitin-ligase function of parkin, one could envision that parkin could have a protective function with respect to AD-related triggers, mainly Aβ peptides and tau proteins, by governing their clearance via enhanced proteasomal-driven degradation [238] or by mitophagy [239].

Several studies indicated that parkin-mediated mitophagy could be altered in AD and that parkin manipulation could potentially rescue AD-like defects. First, Ye and colleagues reported a mitophagic dysfunction in cultured neurons expressing FAD-linked APP mutations as well as in AD-affected brains of patients and that gradual depletion of cytosolic parkin occurs as AD progresses [240]. In agreement with this, the activation of parkin-mediated mitophagy by rapamycin was found to be able to improve cognitive and synaptic plasticity in APP/PS1 mice [241]. Further, Martin-Maestro and colleagues demonstrated that in skin fibroblasts of sporadic AD-affected patients parkin expression was diminished and was accompanied by impaired mitophagy. Of note, parkin overexpression was able to rescue mitophagy failure and lowered ubiquitinated protein loads in these cells [242].

The above studies emphasized the role of parkin in mitophagic defects taking place in AD but did not delineate the precise molecular AD-related targets underlying this dysfunction. A series of studies indicated that both Aβ and Tau could alter parkin-mediated mitophagy and that, conversely, manipulating the mitophagic process could alleviate Aβ- and Tau-related toxicity. First, mitophagy blocks Aβ- and Tau-related pathology in iPSC-derived human neurons as well as in *C-elegans* and mouse models of AD [177]. Interestingly, parkin overexpression restores mitophagy and thereby lowers Aβ-related mitochondrial defects in human cells [243]. In agreement with this, parkin was shown to clear defective mitochondria and ubiquitinated Aβ in the widely used 3xTg-AD triple transgenic mouse model [244]. Further, parkin was shown to prevent cortical atrophy and Aβ-linked brain metabolism defects in this mouse model [245] and reverse intracellular Aβ accumulation in human neuroblastomas and primary cultured neurons [246]. Of utmost importance, parkin overexpression improves hippocampal long-term potentiation and lowers Aβ load in APP/PS1 transgenic mice crossed with mice overexpressing parkin [247].

These above-described models are often overexpressing systems. In order to preclude erroneous conclusions based on an overload of parkin, it was important to note that depletion of endogenous parkin led to the same conclusions. Thus, parkin knock-out cells are more sensitive to intracellular Aβ-induced toxicity [248]. On the other hand, Aβ oligomers were shown to reduce the mitochondrial GTPase Miro1 and trigger autophagy [249], while neurons prepared from parkin-null mice remain resistant to Aβ42-induced toxicity [250]. This set of data altogether indicates an intricate interplay between parkin and Aβ by which parkin could protect cells against Aβ-induced toxicity, while Aβ itself could interfere with parkin-mediated mitophagy.

A parkin-associated phenotype protective against Aβ toxicity has also been indirectly confirmed by pharmacological approaches in various AD-related models. Thus, the neuroprotective vegetal compound β-asarone improved learning and memory in Aβ42-injected rats by increasing PINK1-parkin expressions and lowering Aβ42 loads [251]. This agreed with the decrease in Aβ and augmented autophagy observed in a PC12 cell model [252]. In the same cell line, Panax notoginseng saponins protect against Aβ toxicity by promoting parkin-mediated mitophagy [253]. Finally, nilotinib, an inhibitor of Abl tyrosine kinase, triggers increased autophagic and endogenous parkin levels which are accompanied by Aβ clearance [254].

There exists a network of evidence also indicating that Tau proteins could affect parkin expression and function and that, conversely, endogenous parkin could control Tau. So, intracellular accumulation of Tau, which corresponds to one of the anatomical stigmata in AD brains, is concomitant to mitophagy deficits, as illustrated by increased mitophagy markers. The direct link between these two observations was forged by the demonstration that the overexpression of human Tau induced mitophagy defects in human cells, primary cultured neurons and mouse brains. These changes were accompanied by increased mitochondrial membrane potential and, even more interestingly, by decreased levels of parkin and PINK1 [255]. These observations were corroborated by Cummins and colleagues who confirmed the ability of wild-type as well as a mutant Tau (P301L) to impair mitophagy in neuroblastomas by affecting parkin recruitment to defective mitochondria [256].

Parkin was shown to counteract the alteration of wild-type Tau expression and hyperphosphorylation in human neuroblastoma cells [257]. This could be a general phenotype aspecifically linked to most tauopathies. However, the fact that parkin-associated modification of Tau occurs in the presence of Aβ42 [257] and the recent demonstration that parkin reductions in TS65DN mice (a mouse model of down syndrome [258] in which a triplication of chromosome 21 encoding βAPP occurs in humans) occur before Tau hyperphosphorylation [259] argues in favor of a more selective participation of parkin in Tau modifications taking place in AD. This was corroborated by a series of studies in which the contributions of endogenous parkin to Tau-related pathologies were reported. Thus, in a mouse model in which overexpression of human mutated Tau was combined with *parkin* gene deletion, severe neuropathological alterations were observed, including parkin-dependent selective expression of the mutated Tau transgene in neuronal cell bodies, increased Tau phosphorylation and cerebral atrophy [260]. In this mouse model, the deletion of parkin enhances neuronal lesions in the substantia nigra [261], causes cerebral amyloidosis [262] and triggers aggregation of hyperphophorylated Tau in the cortex and hippocampus [263].

**Figure 1:**
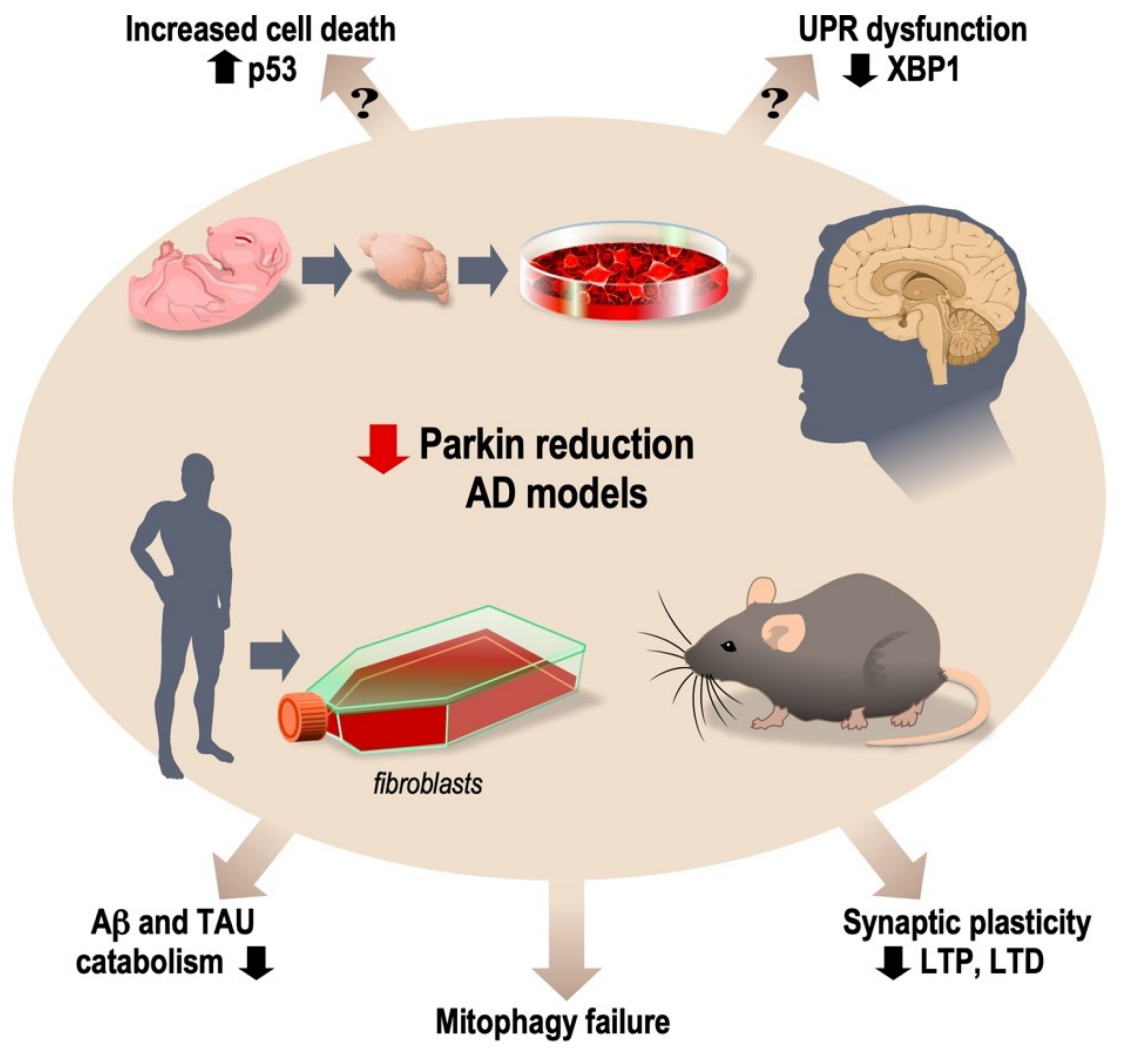
Influence of parkin reduction in XBP1S-linked unfolded protein response (UPR), p53-dependent cell death, long-term potentiation and long-term depression (synaptic plasticity), mitophagy, catabolism of Aβ and Tau proteins in mouse primary cultured neurons as well as in AD-affected human fibroblasts and sporadic AD-affected brains.

Overall, the above-described data strongly support a significant contribution of parkin to AD pathology (Figure 1). This is based on consistent observations derived from multiple cells, animal models and AD-affected brains. Although distinct, these various approaches genuinely identify parkin-mediated contributions to AD, as was supported by a very recent and important study which used a transcriptomic approach to examine the molecular signatures associated with various models of AD. De Bastiani and colleagues analyzed putative analogies and discrepancies between the transcriptomes of three mouse models, namely, 5xFAD, APP/PS1 and human Aβ knocked-in, as well as those of early- and late-onset sporadic cases of AD (De Bastiani et al. bioRxiv, https://doi.org/10.1101/2021.06.09.447404). By means of a regulatory network-based approach aimed at identifying common master regulators (MRs), the authors showed that 5xFAD and APP/PS1 mice shared more MRs with early-onset than with late-onset AD cases, while the hAβ-KI mouse profile did not discriminate between early and late AD cases. However, when considering the 17 MRs overlapping in more than 4 models, only SOX9 and PARK2 were altered across animal/human models. This agreed well with a previous transcriptional analysis delineating PARK2 as a MR in the human AD brain hippocampus [264]. Of note, in De Bastiani et al.’s study, PARK2 appeared to be repressed, thus suggesting a lowering of parkin expression/activity in AD (De Bastiani et al. bioRxiv, https://doi.org/10.1101/2021.06.09.447404). This could be interpreted in several ways. At first sight, the lowering of parkin/ubiquitin ligase activity could be in agreement with decreased Aβ/Tau degradation in AD (see above). On the other hand, parkin is also a transcription factor [204] that can up-regulate presenilin1/γ-secretase [265] and thereby increase Aβ production. Thus, the repression of parkin documented by De Bastiani and colleagues would paradoxically lower γ-secretase-mediated Aβ production. This is not antinomic with the pathogenic process taking place in AD. Thus, we showed that the precursor of Aβ (C99) is very toxic [52], affects many of the cellular pathways altered in AD (see above) [9,14], occurs very early in mice [266] and accumulates in vulnerable neurons in the human brain [267]. Accordingly, we showed that γ-secretase inhibitors potentiate the recovery of intracellular C99 [268] and proposed γ-secretase as a beneficial C99 inactivating enzyme [38]. In this context, the consistent repression of PARK2 and its associated decrease in presenilin1/γ-secretase expression/activity observed in all models examined by De Bastiani and colleagues would yield enhanced levels of C99, in agreement with our C99-centric etiological hypothesis for AD.

The contribution of parkin’s transcriptional function to AD pathology has been further supported by several studies. First, as stated above, besides its ubiquitin-ligase activity, parkin acts as transcription factor [204,269] and up-regulates presenilin 1/γ-secretase [265]. γ-secretase-mediated proteolysis of C99 not only generates Aβ but also the APP intracellular domain (AICD) that acts as a transcription factor [229] able to modulate the expression of several key proteins directly or indirectly contributing to AD pathology [221,231,270,271]. We showed that parkin controls PINK1-linked mitophagy via a presenilin-mediated pathway involving an AICD-FOXO1 functional interaction [189]. Further, it has been shown that the transcription factor XBP1s acts as a key contributor to memory and cognitive functions in AD [272]. Thus, XBP1s is able to rescue hippocampal synaptic plasticity alterations and memory defects in mouse models of AD [273]. As we showed that parkin can control XBP1s activity in a p53-dependent manner [210], one can envision that parkin-mediated improvement in AD-related cognitive defects could, at least partly, involve the control of the XBP1s pathway by parkin.

## 5. Future Perspectives

Besides its well documented contribution to PD, there exists a consistent network of indirect and direct anatomical and functional evidence suggesting that parkin could well contribute to AD pathology. This protein adds to the general picture that proteinopathies harbor common cellular defects and that studying a protein thought to contribute to a given neurodegenerative disease could help to understand another brain pathology. As a corollary, this should open putative novel therapeutic tracks. Sticking to this proposal, parkin could well be envisioned as an additional target to fight the onset and/or progression of AD.

## Abbreviations

AD	Alzheimer’s disease
PD	Parkinson’s disease
ER	Endoplasmic reticulum
PINK1	Phosphatase and TENsin-induced putative kinase 1
XBP1s	Spliced form of X-box binding protein 1
Ab peptides	Amyloid b peptides
PARK	Designated *genes* involved in familial PD
HTRA2	High temperature requirement protein A2
LRRK2	Leucine rich repeat kinase 2
ATP13A2	ATPase type 13A2
ERAD	ER-associated degradation
UPR	Unfolded protein response
ATF6	Activating transcription factor 6
PERK	Pancreatic ER kinase
GRP78	78 Kd Glucose-regulated protein
(IRE1a)	Inositol-requiring enzyme-1 alpha
eIF2 a	Eukaryotic initiation factor 2 alpha
iPSCs	Inducible pluripotent stem cells
PAEL-R	Parkin-associated endothelin receptor-like receptor
OXPHOS	Mitochondrial oxidative phosphorylation system
(ROS)	Reactive oxygen species
MIRO	Mitochondrial Rho
(Drp-1)	Dynamin-related-protein 1
FOXO3a	Forkhead box O3
PEN-2	Presenilin enhancer 2
APH-1	Anterior pharynx-defective 1
